# Aggression Dimensions Among Athletes Practising Martial Arts and Combat Sports

**DOI:** 10.3389/fpsyg.2021.696943

**Published:** 2021-07-09

**Authors:** Karolina Kostorz, Krzysztof Sas-Nowosielski

**Affiliations:** ^1^Department of Theory and Methodology of Physical Education, The Jerzy Kukuczka Academy of Physical Education in Katowice, Katowice, Poland; ^2^Department of Pedagogy, Psychology and Sociology, The Jerzy Kukuczka Academy of Physical Education in Katowice, Katowice, Poland

**Keywords:** judo, karate, aikido, wrestling, fencing, aggression

## Abstract

**Purpose:** The main aim of the research was to analyse aggression dimensions among athletes practising martial arts and combat sports.

**Material and Methods:** There were 219 respondents. The Buss and Perry Aggression Questionnaire (BPAQ) in the Polish adaptation by Siekierka was applied.

**Results:** Martial arts apprentices turned out to present a statistically significantly lower level of hostility (*p* < 0.001) and of the general aggression index (*p* = 0.04) than combat sports athletes. It turned out that lower level of aggression was noted in female participants (physical aggression (*p* < 0.001), verbal aggression (*p* = 0.004), hostility (*p* < 0.001), and the general aggression index (*p* < 0.001). Analysis revealed that the training experience and the training rank did not differentiated the level of the respondents' particular aggression dimensions.

**Conclusions:** It would be advisable to perform parallel analyses in other areas of Poland and take into account the respondents' education and place of residence.

## Introduction

Statistics nowadays indicate an increasing number of crimes involving aggression, such as causing damage to health, engaging in a fight or beating, destroying or damaging property, or robbery crimes, i.e., mugging, extortion, or robbery with violence^1^. Aggression escalation also applies to sport, becoming a subject of growing concern among sports philosophers, pedagogues, and psychologists. It results in progressive dehumanisation and devaluation of sport, manifested in violence on sports pitches and stands (Cynarski and Litwiniuk, 2007; Vertonghen and Theeboom, 2010; Chahal and Chaudhary, 2012). It is worrying that aggression, even under the demands of rules and regulations, sometimes becomes the predominant source of players' excitement, pleasure, and satisfaction, or even the key motive for participating in sports (Krishnaveni and Shahin, 2014). Moreover, aggression combined with the desire to win often pushes athletes into breaching the principles of noble competition and makes fair play forgotten (Graczyk et al., 2010; Vertonghen and Theeboom, 2010; Krishnaveni and Shahin, 2014). Transferring the views of Bandura (2001) to the sports context, one could suppose that if athletes' aggressive behaviours are not punished or, worse, become rewarded (resulting in scoring a point, scoring a goal, winning a medal, or preventing the^1^ rival from doing so), they will be frequently observed during competitions (Bukowska et al., 2012; Chahal and Chaudhary, 2012). Bukowska et al. (2012) indicate that taking up physical activity can become a way to “clear oneself” of aggression in a socially accepted way. On the other hand, it can reinforce the tendency to react with more aggression in everyday life and strengthen the character traits that are useful in sports competition, but not necessarily in social life, such as bravery or obstinacy (Szmajke and Doliński, 1991; Vertonghen and Theeboom, 2010; Tomczak, 2013). The research conducted so far to investigate this problem has not yielded clear results. In view of the above, it is important to carry out further detailed studies.

It should be emphasised that in its majority, aggression observed in athletes' actions is instrumental in nature: the aim is to achieve non-aggressive benefits by means of aggressive behaviours (Mroczkowska et al., 2008; Klimczak et al., 2014; Martinkova and Parry, 2016). In sport, manifestations of uncontrolled aggression are undesirable for two reasons. They provide young people with negative behavioural patterns, at the same time distorting the image of sport (Mroczkowska et al., 2008), as well as increase the risk of a career-ending injury (Pedersen, 2007). In the case of combat sports, considering the propensity for violent behaviours is not only of cognitive significance because of the dangers associated with the athletes' extensive skills and strength, which can be employed outside the sports arena to use violence, engage in fights, and commit various crimes (Reynes and Lorant, 2001, 2002a,b, 2004; Graczyk et al., 2010; Klimczak et al., 2014; Martinkova and Parry, 2016; Harwood et al., 2017).

The awareness of the factors mentioned above, the inconsistencies in the results of previous studies, were inspirations to conduct our own investigations. There is no doubt that the goals, tasks, and other aspects related to sports competition and recreational practise of traditional martial arts are divergent (Figueiredo, 2009; Martinkova and Parry, 2016), so it was deemed reasonable to analyse aggression dimensions among people practising martial arts and combat sports.

## The Purpose of the Study

The objective of the study was to determine whether there exists a difference with regard to aggression dimensions between respondents and the normative value (Tucholska, 1998), as well as between combat sports fighters and athletes practising martial arts and with the consideration of the training period and degree, and the respondents' gender.

The following research questions were submitted:

1. Is there a statistically significant difference in aggression dimensions between respondents and the normative value developed by Tucholska (1998)?
2. Is there a statistically significant difference in aggression dimensions between athletes practising martial arts and combat sports?
3. Do the factors of gender, training experience, and training rank determine the results of the studied variables, i.e., physical aggression, verbal aggression, angry, hostility and the general aggression index?

Due to the exploratory nature of the research, it was decided to abandon the research hypotheses.

It was expected that the analyses would significantly broaden the scope of the existing knowledge on the subject, thus allowing to obtain important results, especially in the field of humanities, i.e., psychology, sociology, pedagogy, and ethics. Besides, many authors (Tiric-Campara et al., 2012; Kuśnierz and Bartik, 2014; Martinkova and Parry, 2016; Harwood et al., 2017; Basiaga-Pasternak et al., 2020) indicate the scarcity of publications on martial arts and combat sports. It should also be added that the issue of aggression has been extremely rarely raised, especially in the last decade. Undoubtedly, all the above aspects also became an inspiration to perform own analysis.

## Materials and Methods

### Participants and Procedure

The research was performed between March 2017 and November 2017 in sports clubs and sections of martial arts (Pszczynska Martial Art, capoeira, and aikido) and combat sports (judo, wrestling, fencing, taekwondo, and Kyokushin karate) in the Silesia Province in Poland. Nonprobability consecutive sampling was applied in the analyses. After obtaining permissions and support from the club or organisation representatives, researcher approached athletes. Researcher invited potential participants and presented the study aims and conditions. Informed consents were obtained. Those who agreed to participate in the study got the questionnaire with a request to fill it. The respondents were of both genders, at least 15 years old; they had been training martial arts or combat sports for at least 1 year. All individuals submitted a written consent to voluntarily participate in the research. In the case of minors, the consent was obtained from the parents or legal guardians. Over 550 questionnaires were distributed, 243 were collected back. However, some questionnaires were not filled in completely, and these were excluded from consideration. Owing to the above mentioned inclusion criteria and conditions, and lack of complete documentation, results obtained from 219 respondents underwent final analysis. Out of the participants, 113 (51.60%) practised martial arts and 106 (48.40%) were combat sports athletes. Women (*n* = 101) constituted 46.12% of the study group. Their mean age equalled 23.61 years (*SD* = 4.83). Men (*n* = 118) constituted 53.88% of the study group, with the mean age of 24.31 years (*SD* = 5.91). In the analyses, the respondents were divided into two groups depending on training experience: those who had been training for less (107 people) and for more than 5 years (112 athletes). The study also involved analyses depending on the participants' training rank. It was assumed that a high rank meant having at least 3 kyu (3 kup for taekwondo practitioners), and a white and orange colour for capoeira practitioners. In the case of martial arts styles whose representatives took part in the research, this rank is achieved, on average, by individuals after 2.5–3.5 years of regular training. The trainees have high technical skills; they are involved by instructors in conducting parts of the classes, e.g., warm-ups, stretching, etc., and also act as assistants. For combat sports such as fencing and wrestling, the rank of a player was determined by the coach prior to the respondent's completing the questionnaire. The coaches were acquainted with the criteria applied in sporting level assessment, i.e., scores of the official ranking list and the sporting classes awarded for strictly defined achievements. In the statistical analyses, “high rank” referred to respondents who obtained very good (medal zone) or good (places 4–15) sports results in their age categories and achieved a regular increase in their sporting level. These competitors are already included in a specialised training phase. With these criteria, eventually, 94 subjects, including 43 combat sports athletes, had a low rank, and 125 individuals, including 62 martial arts practitioners, had a high rank.

The consent for conducting the research was obtained from the Bioethical Committee of the Academy of Physical Education in Katowice.

### Instruments

The diagnostic poll method with the questionnaire technique served to fulfil the assumed aims. A standardised research tool was applied. Aggression was assessed with the Buss-Perry Aggression Questionnaire (BPAQ) (Buss and Perry, 1992) in the version developed by the Amity Institute in Warsaw, Poland (Siekierka, 2005). The tool contains 29 statements designed to evaluate four dispositional components of aggression: physical aggression (e.g., I have threatened people I know.), verbal aggression (e.g., My friends say that I'm somewhat argumentative.), anger (e.g., Some of my friends think I am a hothead.), and hostility (e.g., Other people always seem to get the breaks.). The results for these subscales total the general aggression index of an individual. The task of the respondent was to rank the particular statements using a five-point Likert scale, where 1 meant “does not match my personality at all” and five stood for “fully matches my personality.” The questionnaire is characterised by a clear theoretical model and satisfactory psychometric properties (Archer, 2004; Siekierka, 2005; Aranowska and Rytel, 2012). In the study conducted in Poland by Aranowska and Rytel (2012) exploratory and confirmatory models were evaluated among a sample of 3,990 Polish participants (aged from 10 to 79 years). A five-factor structure resulted in the exploratory analysis and five-factor structure showed acceptable fits in confirmatory analyses (with the Cronbach's α ranging from 0.60 for angry to 0.87 for general aggression index).) Measures used to assess the fit of the model to the data took acceptable values, i.e., Root Mean Square Error of Approximation—RMSEA = 0.06; Comparative Fit Index—CFI = 0.86, Normed Fit Index—NFI = 0.85 (Aranowska and Rytel, 2012). Aranowska and Rytel (2012) showed appropriate to high reliabilities for all scales, i.e., Cronbach's α was 0.85 for physical aggression, 0.72 for verbal aggression, 0.83 for angry, 0.77 for hostility, and 0.89 for whole items. In own research, it was obtained indicators of the questionnaire reliability with the Cronbach's α ranging from 0.75 for physical aggression to 0.81 for verbal aggression (Table 1). So, it turned out that all scales had satisfactory reliability (John and Benet-Martines, 2000).

**Table 1:** Skewness, kurtosis and Cronbach's alpha values.

**Studied variable**	***As***	***Me***	***Mo***	***V***	***Ku***	***Cronbach's alpha***
Anger	0.17	19.00	19.00	4.71	0.67	0.77
Physical aggression	−0.08	21.00	21.00	9.58	−1.11	0.75
Hostility	−0.60	22.00	23.00	7.65	0.57	0.76
Verbal aggression	0.42	15.00	15.00	5.58	1.04	0.81
General aggression index	−0.22	77.00	78.00	4.88	−1.16	0.79

*Source: the authors' own research*.

The tool also included information for the participants describing the aim and subject of the research, as well as a list of demographic questions, which allowed to obtain data on the respondents' age, gender, training experience, and rank.

### Statistical Analysis

The basic analysis of the data employed descriptive statistics for the whole sample, for the groups of martial arts practitioners and combat sports athletes, as well as for the population stratified by gender, training experience, and training rank. The mean (*M*), standard deviation (*SD*), median (*Me*), mode (*Mo*), coefficient of variation (*V*), and skewness (*As*) were calculated. Kurtosis (*Ku*) was used to measure concentration in the study. The distribution was tested for normality with the Shapiro-Wilk W test. Levene's test served to assess the equality of variances. The nature of the variable distributions, Levene's test results, and the sample size (*n* = 219) allowed the use of parametric tests to verify the significance of differences between the tested variables (significance tests for differences in independent samples). The significance level was assumed at *p* < 0.05. In order to compare the levels of the aggression dimensions among the practitioners of martial arts and combat sports with the normative value developed by Tucholska (1998), a significance test called ‘Difference between Two Means' was applied, available in the Statistica software. The effect size (*ES*) was calculated (Cohen's *d*) not only whenever statistically significant differences between the examined variables were revealed. It was assumed to be small for values ranging 0.2–0.49, moderate for values ranging 0.5–0.79, and large for *ES* > 0.79 (Rodriguez, 2007).

Based on the data available on the website of the Central Statistical Office in 2018^2^, it was assumed that about 101,774 people practise martial arts or combat sports in Poland. Therefore, it has been calculated^3^ that 383 is the minimum recommended size for the own study. At the same time, it turned out that for the sample of 219 respondents, the margin of error was 6.62%. It should be added that in the case of 219 people participating in the own research, the confidence level above 86% was obtained.

The analyses were performed with Microsoft Office Excel 2010 and StatSoft Statistica v. 12.

## Results

The study revealed cases of asymmetric (skewed) distributions. However, all variables fell within the range of < -1, 1>, both for the whole sample and irrespective of the type of physical activity undertaken by the respondent, their gender, training experience, and training rank. Thus, it was assumed that the distribution of the studied variable was moderately asymmetric. The kurtosis (*Ku*) for all variables in each group fell within the < -2, 2> range, indicating that the concentration around the mean value was satisfactory (Table 1).

In the second step of the analysis, the results of the respondents were compared with the normative value developed by Tucholska (1998) (Table 2).

**Table 2:** Comparison of the level of aggression dimensions between the respondents' results and the normative value.

**Gender**	**Studied variable**	**Martial arts and combat sports practitioners**		**Normative value**		***p*****-value**		**Effect size**
		***M***	***SD***	***M***	***SD***	**One-tailed test**	**Two-tailed test**	
Women	Anger	19.07	4.05	19.2	4.9	0.42	0.84	0.03
	Physical aggression	18.72	4.26	18.9	5.7	0.40	0.80	0.04
	Hostility	19.52	4.15	21.8	4.7	0.002	0.004	0.52
	Verbal aggression	14.06	2.90	14.5	2.9	0.14	0.29	0.15
	General aggression index	71.38	11.66	74.3	13.4	0.05	0.10	0.23
Men	Anger	19.24	3.78	19.1	4.6	0.41	0.81	0.03
	Physical aggression	22.62	4.29	22.9	6.4	0.35	0.71	0.05
	Hostility	22.25	4.15	23.6	5.1	0.02	0.04	0.30
	Verbal aggression	15.10	2.38	15.7	3.0	0.06	0.11	0.23
	General aggression index	79.20	11.86	81.4	14.8	0.12	0.24	0.17

*Source: the authors' own research, with reference to Tucholska (1998)*.

A statistically significantly lower level of hostility, and, in the case of the female gender, also of the general aggression index (*p* = 0.05 in one-tailed test; *ES* = 0.23), was observed among women (*p* = 0.002 in one-tailed test; *p* = 0.004 in two-tailed test; *ES* = 0.52 and among men (*p* = 0.02 in one-tailed test; *p* = 0.04 in two-tailed test; *ES* = 0.30) training martial arts and combat sports.

The following analyses considered the type of the undertaken physical activity (Table 3).

**Table 3:** Comparison of the level of aggression dimensions between martial arts and combat sports athletes.

**Studied variable**	**Martial arts**		**Combat sports**		***t***	***df***	***p*-value**	**Effect size**
	***M***	***SD***	***M***	***SD***				
Anger	19.09	3.87	19.24	3.95	0.28	217	0.78	0.04
Physical aggression	20.38	4.71	21.29	4.65	1.44	217	0.15	0.20
Hostility	19.99	4.28	22.06	4.22	3.60	217	<0.001	0.49
Verbal aggression	14.45	2.67	14.80	2.69	0.97	217	0.33	0.13
General aggression index	73.91	12.63	77.39	11.95	2.09	217	0.04	0.28

*Source: the authors' own research*.

Martial arts apprentices turned out to present a statistically significantly lower level of hostility (*p* <0.001; *ES* = 0.49) and of the general aggression index (*p* = 0.04; *ES* = 0.28) than combat sports athletes.

It was very important to implement analyses with the consideration of the respondents' gender (Table 4).

**Table 4:** Comparison of the level of aggression dimensions between women and men.

**Studied variable**	**Women**		**Men**		***t***	***df***	***p*-value**	**Effect size**
	***M***	***SD***	***M***	***SD***				
Anger	19.07	4.05	19.24	3.78	−0.32	217	0.75	0.04
Physical aggression	18.72	4.26	22.62	4.29	−6.72	217	<0.001	0.91
Hostility	19.52	4.15	22.25	4.15	−4.83	217	<0.001	0.66
Verbal aggression	14.06	2.90	15.10	2.38	−2.92	217	0.004	0.40
General aggression index	71.38	11.66	79.20	11.86	−4.91	217	<0.001	0.66

*Source: the authors' own research*.

It turned out that only the level of anger (*p* = 0.75) did not differentiate women and men. In the remaining cases, a lower level of aggression was noted in female participants. What draws attention is the effect size, which was large with regard to physical aggression (*ES* = 0.91; *p* < 0.001), small in the case of verbal aggression (*ES* = 0.40; *p* = 0.004), and moderate for hostility (*ES* = 0.66; *p* < 0.001) and the general aggression index (*ES* = 0.66; *p* < 0.001).

To answer the question whether the level of aggression dimensions depended on the respondents' training experience or training rank, a significance test for differences in independent samples was applied (Tables 5, 6).

**Table 5:** The level of aggression dimensions depending on the training experience.

**Studied variable**	**>** **5 years of experience**		** <5 years of experience**		***t***	***df***	***p*-value**	**Effect size**
	***M***	***SD***	***M***	***SD***				
Anger	19.10	3.92	19.22	3.90	−0.24	217	0.81	0.03
Physical aggression	21.38	4.74	20.24	4.58	1.79	217	0.07	0.25
Hostility	21.44	4.29	20.52	4.40	1.56	217	0.12	0.21
Verbal aggression	14.92	2.73	14.31	2.60	1.70	217	0.09	0.23
General aggression index	76.83	12.55	74.30	12.12	1.52	217	0.13	0.21

*Source: the authors' own research*.

**Table 6:** The level of aggression dimensions depending on the training rank.

**Studied variable**	**High rank**		**Low rank**		***t***	***df***	***p*-value**	**Effect size**
	***M***	***SD***	***M***	***SD***				
Anger	19.47	3.82	18.74	3.95	−1.37	217	0.17	0.19
Physical aggression	21.27	4.48	20.22	4.81	−1.64	217	0.10	0.23
Hostility	21.45	4.45	20.38	4.25	−1.80	217	0.07	0.25
Verbal aggression	14.61	2.75	14.64	2.64	0.08	217	0.93	0.01
General aggression index	76.80	11.53	73.99	12.89	−1.67	217	0.10	0.23

*Source: the authors' own research*.

The presented data imply that neither the training experience nor the training rank differentiated the level of the respondents' particular aggression dimensions.

## Discussion

It turned out that in comparison with the normative value developed by Tucholska (1998), a statistically significantly lower level of hostility, and, in the case of the female gender, also of the general aggression index, was observed among women and among men training martial arts and combat sports. The obtained results of own analyses are in line with other authors' outcomes (Wojdat et al., 2017a), who also noticed that regardless of the respondents' gender, the hostility in athletes was lower as compared with the average for the Polish population. Furthermore, a lower level of hostility to the environment was observed in karate, aikido, and taekwondo practitioners (Graczyk et al., 2010). In the same research, only boxers did not reveal a favourable effect of sport on this aggression dimension. These findings corroborate the earlier views of Graczyk (1994). It is worth emphasising that a lower level of hostility is beneficial for health, as confirmed by the research conducted in Finland by Sawicki et al. (2015). It turned out that the sense of hostility was associated with an increased risk of myocardial infarction and stroke, as well as the development of atherosclerotic diseases and diabetes. Additionally, people with high levels of this aggression dimension receive much less social support and face more negative attitudes in their working environment, which contributes to experiencing even more grief, uncertainty, injustice, and suspiciousness. The lower values of this aggression dimension determined both in the presented research and in reports by other authors (Wojdat et al., 2017a) indicate that practising combat sports, mainly traditional martial arts, can satisfy the need for belonging to a social group and contribute to the feeling of confidence about the environment and to showing a friendly attitude to others, mutual cordiality, and kindness. Supinski (1991), after: Tomczak (2013) observed that karate and judo athletes, as well as boxers presented a statistically significantly lower level of aggression than female students of one of the secondary schools in Wrocław. Furthermore, Daniluk et al. (2004) found that national team judo athletes demonstrated low and moderate degrees of aggression as compared with the general population. Budnik (2004) revealed that karate practitioners were characterised by lower aggression intensity than students. In addition, Kuśnierz and Bartik (2014) proved that the practise of combat sports has a favourable impact on fighters; even though ju-jitsu fighters achieved the highest level of physical and verbal aggressions in groups under research, the general level of their aggression was lower than in the case of non-training persons. Mroczkowska et al. (2008) also noticed that in the case of wrestlers, judo athletes, and traditional karate practitioners, the general aggression index was far below the maximum outcome. It should be mentioned, too, that Wojdat et al. (2017a) observed a statistically significant relationship between education and a decrease in general aggression among judo athletes, regardless of gender, with a high determination indicator value. In the control group, this correlation was not implied (Wojdat et al., 2017b).

On the other hand, Szmajke and Doliński (1991) mention the results of studies showing that young people who train ‘high contact' sports present a larger tendency toward aggressive behaviours, both in sport and in everyday life, than individuals with no such experience. Jasiński et al. (2002) also noticed that the level of anxiety and aggression was statistically significantly higher in training youth than among young people who had never practised hand-to-hand combat. Other authors also observed that contact sports fighters are more aggressive than contestants in non-contact sports (Chahal and Chaudhary, 2012; Kumar, 2015; Rui and Cruz, 2017). The results of own analyses are also not in line with research conducted by Tomczak (2013). He observed that women and men who practised wrestling or fencing, as well as female judo competitors achieved lower levels of aggression compared with the normative value. In the same study, karate competitors and foil fencers obtained results similar to those of the standardisation group respondents. Tomczak (2013) proved that on exceeding certain values, aggression may even interfere with effective functioning. The author noted negative correlations between physical aggression and the frequency of manifesting the style of planned actions both in judo competitors and in female foil fencers, the style of various actions in female foil fencers, and the effectiveness of sports action in male foil fencers. Tomczak (2013) also indicated that respondents with a higher level of hostility manifested a less diverse style than athletes with a lower average score in this variable. According to Tomczak (2013), a lower level of aggression has more significance in very precise disciplines, such as foil fencing, where special control is required. Furthermore, Wrześniewski (2015) revealed that taekwondo practitioners obtained higher levels of all aggression dimensions than the control group, consisting of physical education and physiotherapy students.

Own research showed that martial arts apprentices presented a lower level of hostility and of the general aggression index than combat sports athletes. Moreover, considering gender and type of the undertaken physical activity, it also turned out that both women and men practising combat sports obtained higher levels of hostility than martial arts respondents. Szmajke and Urbanowicz (2012) indicate that the tendency toward competition is not an inherent trait of human nature but a very strong instinct and that the necessity to engage in competition leads to negative social consequences. According to Aronson (1995), rivalry is not only among the main causes of inter-group prejudices and hostility, of a decline in openness and trust in relationships, but also constitutes a factor strengthening these phenomena. It is pointed out that the very expectation of competition usually leads to attributing negative features, reprehensible motives for action, and biassed judgments to the opponent (Doliński and Szmajke, 1991; Szmajke and Urbanowicz, 2012). What is more, undertaking physical activity, as mentioned in the introduction, can reinforce the tendency to react with more aggression and fury in everyday life (Szmajke and Doliński, 1991). Thus, exercise can strengthen the character traits that are useful in sports fight, but not necessarily in social life. One might suppose that socialisation of anxiety and aggression behaviours based on rigid rules and principles of martial arts statistically significantly decreases their incidence in athletes. These views have been confirmed in publications by many authors (Jasiński et al., 2000; Lu, 2008; Mroczkowska et al., 2008; Krishnaveni and Shahin, 2014; Harwood et al., 2017). Kubacka-Jasiecka and Wrześniewski (2012) claim that although athletes practising martial arts for recreational purposes or participating in combat sports competitions have to self-induce a certain degree of aggression, they do not differ in this respect from individuals engaged in other sports disciplines or physical activities. These authors found average levels of aggression among aikido, karate, judo, taekwondo, and Krav Maga practitioners. Basiaga-Pasternak et al. (2020) proved that competitive and non-competitive combat sports athletes had similar level of all types of aggression. The obtained own results are in line with a study carried out by Budnik (2005), who observed a larger tendency to manifest aggression in sports karate competitors than in respondents practising the traditional form of this discipline. Mroczkowska et al. (2008) also indicated that the general aggression index was statistically significantly lower in people practising traditional karate than in judo athletes and wrestlers. It is worth adding that higher sociability, impulsiveness, and hostile aggression were observed among female basketball players than in female taekwondo practitioners (Litwiniuk and Daniluk, 2009). One should also refer to a study by Graczyk et al. (2010), who found a higher average level of emotional self-aggression in boxers and aikido competitors than in karate and taekwondo athletes. They noted the biggest mean indices of physical self-aggression, hostility to the environment, unconscious aggressive inclinations, as well as displaced and indirect aggression among boxers. These results are very close to those obtained in a prior research performed by Graczyk (1994). Besides, Mazur and Organista (2015) showed that women practising kickboxing and wrestling achieved statistically significantly higher levels of physical aggressiveness than female respondents practising artistic gymnastics and synchronised swimming. The same research revealed that the difference in the total aggression index remained at the trend level, although it should be mentioned that women practising kickboxing and wrestling were slightly more aggressive again. The authors also noted that female wrestling competitors were characterised by a statistically significantly higher average level of suspiciousness and the feeling of guilt than women practising kickboxing. In addition, Vertonghen et al. (2014) found higher levels of physical aggression among kickboxing and Thai boxing athletes as compared with people practising judo, aikido, and karate. In turn, Kuśnierz et al. (2014) did not observe statistically significant differences in physical aggression among capoeira practitioners, boxers, and ju-jitsu competitors. It is worth emphasising that the lowest score was shown in respondents training capoeira, and the biggest average referred to both boxers and the control group, which consisted of high school and physical education students. The authors indicate that contemporary boxers are more prone to aggressive behaviours than previous generations undertaking this type of physical activity. Kuśnierz et al. (2014) proved that the highest mean value for verbal aggression was obtained by non-training respondents, and the lowest by capoeira practitioners. The highest level of anger was noted in the control group, and the lowest in ju-jitsu athletes. In turn, the highest level of hostility was found in the control group and boxers, and the lowest in ju-jitsu competitors. The biggest mean general aggression index was reported for the control group, and the smallest in ju-jitsu athletes. A study carried out by Piepiora et al. (2016) is also worth mentioning. The authors observed that practising different styles of karate, with different rules of kumite, did not differentiate competing athletes in terms of aggressiveness. They noticed that karate practitioners fighting in the semi-contact system (Shotokan karate) presented substantially lower levels of the general aggression index, physical and verbal aggression, and suspiciousness than other competitors in kumite systems. Karate athletes competing in mixed fighting (Shidokan karate) were characterised by significantly higher levels of physical aggression and irritability than those engaged in less violent forms of kumite. Knockdown karate (Kyokushin karate) practitioners tended to have significantly higher overall levels of aggression, both physical and verbal, and suspiciousness than representatives of the semi- contact system (Shotokan karate). Mixed fighting (Shidokan karate) athletes turned out significantly less physically aggressive and irritable than full contact (Oyama karate) fighters and relatively more negative and resentful than those competing in knockdown (Kyokushin karate). It should be emphasised that all the results for competitors and students are presented as average indicators of aggression.

As expected, the own analyses revealed that only the level of anger did not differentiate women and men. In the remaining cases, a lower level of aggression was noted in female participants. What draws attention is the effect size, which was large with regard to physical aggression, small in the case of verbal aggression, and moderate for hostility and the general aggression index. In turn, Wojdat et al. (2017a) noted that among judo athletes, women obtained a statistically significantly lower average only with reference to physical aggression as compared with men. There is no doubt that aggression, especially physical aggression, is one of the functional dimensions for which differences between women and men are indisputable and have been regularly reported in the literature since the 1820s (Hyde, 2005; Rytel, 2011). The observed differences can be explained by biological, social, and evolutionary factors. They can result from the processes of learning aggression; according to the social role theory, gender differences are insignificant in early childhood but they become gradually intensified during childhood as a consequence of the gender role learning process (Rytel, 2011). In addition, as implied by the sexual selection theory, rooted in sociobiology and evolutionary psychology (Rytel, 2011), differences in the level of aggression should emerge early in ontogenesis and reach their highest values in young adults (aged 20–30 years), i.e., at the peak of the reproductive capacity (Archer, 2004, 2009). With reference to the former theory, the higher level of physical aggression in men than in women is associated with the requirements of the social role, while in the case of the latter, it results from the competition in the access to female partners, entailing the need to apply more risky strategies to guarantee a reproductive success. Both theories additionally assume that there are larger differences between males and females in physical than in verbal aggression (Rytel, 2011). The results of our own analyses related to verbal aggression can also be explained by referring to study by Rytel (2011). These authors indicate that for women, the language of conversation is primarily a means to communicate, build relationships, and create bonds. For men, in turn, talking is a way to remain independent and to gain and maintain position in the hierarchical social order. Our results are in line with research performed by Buss and Perry (1992), as well as with the meta-analysis conducted by Archer (2004), which revealed that most studies did not report statistically significant inter-gender differences in the anger variable. Exceptions included a study carried out in England in which a higher average value was observed in women, and an Australian one that revealed an escalation in the aggression dimension among men. It should be noted that Archer (2004) did not provide results concerning the level of hostility. The results of own research do not corroborate the studies in which no statistically significant differences were found between women and men for the aggression dimension (Nakano, 2001; Gerevich et al., 2007). The results of own research are partly in line with a study conducted by Rytel (2011).

Taking into account training experience, own results do not coincide with the outcomes obtained by other authors. Kurpel et al. (2005) observed that in wrestlers, aggression decreased with an increase of competition experience. According to Wrześniewski (2015), practising taekwondo reduces aggressive tendencies, but only after several years of training. Drumińska et al. (2016) revealed a trend toward lower general aggression with increasing training experience in children aged 12–15 years practising judo, taekwondo, and karate. However, in taekwondo athletes, positive correlations were observed between training experience and all aggressive tendencies except physical aggression (Zalech, 2002), and the strongest relationship was demonstrated in the case of verbal aggression. Budnik (2004) noted that karate practitioners with longer training experience were characterised by a higher level of indirect aggression and suspiciousness than athletes with a shorter sporting career. Basiaga-Pasternak et al. (2020) noticed that despite the lack of statistical significance it was noted that the level of almost all types of aggression was higher in the amateurs than in the professionals. It is worth referring to a study performed by Wojdat et al. (2017a), who found a statistically significant relationship between the general aggression index and training experience among judo practitioners. However, it turned out that this correlation was negative in women and positive in men. It is suggested that an increase in the aggression level among athletes with longer training experience can result from frustration caused by lack of sports successes.

Regardless of gender, the own analyses revealed that neither the training period nor the training rank differentiated the level of the respondents' particular aggression dimensions. The obtained results are not in line with studies by other authors, who noted, for example, that practising judo decreased or did not influence the level of aggression (Lamarre and Nosanchuk, 1999; Nosanchuk and Lamarre, 2002). In turn, Reynes and Lorant (2001, 2002a,b); Reynes and Lorant (2004) observed an increase in aggression during judo training and its decrease as a result of karate training. According to these authors, practising combat sports can have a beneficial effect on aggression dimensions, but this can be demonstrated only after a longer period of training; in 10–12-year-old boys, 1 year's training did not yield the desired results (Reynes and Lorant, 2002a,b). Ziaee et al. (2012) implied that karate training had a positive impact on controlling aggression among adolescents, but no such influence was found in respondents practising judo. Attention should also be paid to the worrying results of a study by Rotter et al. (2015), in which a statistically significantly higher mean value for physical aggression was obtained among young people practising sports 3–4 times a week than in their less physically active peers. Among judo and wrestling competitors, a higher level of aggression was observed in athletes from the national team as compared with individuals at a lower training level (Zyto-Sitkiewicz, 1986), which is not in line with research carried out by Skelton et al. (1991), or Daniels and Thornton (1992). It has been indicated that sport engages people with a higher tendency toward physical aggression, and this aggression can only be alleviated with a strong sporting commitment (Reynes and Lorant, 2002a,b; Wojdat et al., 2017b).

On the basis of the results obtained in our own analyses and the research of other authors, it can be stated that aggressive behaviour can be changed, modified, and relieved, but also accumulated through physical activity (Mroczkowska et al., 2008; Vertonghen and Theeboom, 2010; Tiric-Campara et al., 2012; Kuśnierz et al., 2014; Martinkova and Parry, 2016; Harwood et al., 2017). So, these findings provide an important basis to understand personality differences in aggressive-related variables, e.g., assess competitive state anxiety, the level of emotional intelligence, temperamental characteristics.

## Conclusions

Our research allows us to formulate the following conclusions:

1. As compared with the normative value (Tucholska, 1998), both women and men training martial arts and combat sports presented lower levels of hostility; in the case of females, this also applied to the general aggression index.
2. Lower values for hostility and the general aggression index were noted among those practising martial arts than in combat sports athletes.
3. Women demonstrated lower levels of all aggression dimensions, with the exception of anger, for which the difference turned out statistically insignificant.
4. The duration and level of training did not determine the degree of aggression in the respondents.

On the basis of the results obtained in our own analyses it can be stated that regular and with full commitment training combat sports and especially martial arts supervised by a trainer who pays attention to educational aspects and compliance with the rules can become a method of reducing the level of hostility. Martial arts with a centuries-long tradition are regarded in social opinions as a carrier of many desired, moral values and function as an education system for children and young people, not only in Eastern societies. On the basis of the own study, it should be noticed that it is worth introducing elements of martial arts into school physical education and as part of organised physical activity in free time.

It should also be noted that the levels of particular aggression dimensions are not constant, but characterised by a certain dynamics and changeability in a lifetime. The consciousness of these phenomena makes one thus approach any results obtained in a given moment with considerable caution. The results cannot be perceived as final conclusions. Also, because of the sample size, the research presented here should constitute, among others, a starting point for further considerations.

Among the possible areas of expanding this research project, performing longitudinal studies with the application of a cross-sectional and sequential analysis design seems justified. They would aim at determining the changes occurring in the motivational processes, the accepted hierarchy of values, as well as aggressive tendencies, e.g., during a 2-years training cycle. Moreover, as the presented research involved only sports clubs and sections of martial arts and combat sports located in the Silesia Province, it would be advisable to perform parallel analyses in other areas of Poland. It seems that taking into account the respondents' education and place of residence (as divided into the city and the country) would contribute to a more expanded analysis. In this way, the exploration of the subject and the study conclusions could become more comprehensive and more valuable.

## Data Availability Statement

The raw data supporting the conclusions of this article will be made available by the authors, without undue reservation.

## Ethics Statement

The studies involving human participants were reviewed and approved by the Bioethics Committee of the Academy of Physical Education in Katowice (KB/04/2017 as of 13 February 2017). Written informed consent to participate in this study was provided by the participants' legal guardian/next of kin.

## Author Contributions

All authors listed have made a substantial, direct and intellectual contribution to the work, and approved it for publication.

## Conflict of Interest

The authors declare that the research was conducted in the absence of any commercial or financial relationships that could be construed as a potential conflict of interest.

## Footnotes

^1^Available online at: https://statystyka.policja.pl/st/informacje/57720,Przestepczoscmlodych-ludzi-zwiazana-z-agresja.html

^2^Available online at: https://stat.gov.pl/files/gfx/portalinformacyjny/pl/defaultaktualnosci/5495/13/2/1/kultura_fizyczna_w_polsce_w_2018.pdf

^3^Available online at: http://www.raosoft.com/samplesize.html
